# Speciation Analysis of Arsenic Compounds by HPLC-ICP-MS: Application for Human Serum and Urine

**DOI:** 10.1155/2018/9462019

**Published:** 2018-11-13

**Authors:** Manh Ha Nguyen, Tien Duc Pham, Thi Lien Nguyen, Hai Anh Vu, Thi Thao Ta, Minh Binh Tu, Thi Hong Yen Nguyen, Dinh Binh Chu

**Affiliations:** ^1^Faculty of Chemistry, VNU University of Science, Vietnam National University-Hanoi, 19 Le Thanh Tong, Hoan Kiem, Hanoi 100000, Vietnam; ^2^National Institute of Hygiene and Epidemiology, 1 Yersin, Hai Ba Trung, Hanoi 100000, Vietnam; ^3^School of Chemical Engineering, Hanoi University of Science and Technology, 1 Dai Co Viet, Hai Ba Trung, Hanoi 100000, Vietnam

## Abstract

A high-performance liquid chromatography (HPLC) in combination with inductively coupled plasma mass spectrometry (ICP-MS) as an elemental specific detector was used for the speciation analysis of arsenic compounds in urine and serum samples from Vietnam. Five arsenic species including arsenite (As^III^), arsenate (As^V^), monomethylarsonic acid (MMA), dimethylarsinic acid (DMA), and arsenobetaine (AsB) were studied. A gradient elution of ammonium carbonate ((NH_4_)_2_CO_3_), ethylenediaminetetraacetic acid disodium salt (Na_2_EDTA), and methanol at pH 9.0 utilizing Hamilton PRP-X100 strong anion-exchange column allowed the chromatographic separation of five arsenic species. In this study, urine and serum samples were prepared by dilution in solvent and protein precipitation by trichloroacetic acid, respectively. The extraction efficiency was greater than 91% for urine matrix, and recoveries from spiked samples were in the range of 94–139% for the arsenic species in human serum. The method limit of detection (MDL) and limit of quantification (MQL), which were calculated by signal to noise ratio, were found to be 0.3–1.5 and 1.0–5.0 ng·mL^−1^, respectively. The concentration of arsenic species in 17 pairs of urine and serum samples from Vietnam was also quantified and evaluated. The major species of arsenic in the urine and serum samples were AsB and DMA.

## 1. Introduction

Arsenic (As) contamination in groundwater and its potential health effects have been a critical issue and received considerable attention in many regions around the world. The toxicities of inorganic arsenic compounds have been reported in certain areas and have caused a large number of serious health issues [1, 2]. In order to understand the status of As contamination in water and human exposure, the most common approach is determining the total As concentration. However, speciation analysis provides a more detailed picture of exposures to different forms of arsenic, and thus, it improves understanding of arsenic toxicity. The differences in the sensitivity to inorganic As-related diseases among individuals have been reported, indicating the possible association with individual variations in inorganic metabolism [3]. The methylation of inorganic As or DMA as endpoint related to As was also reported [4]. Therefore, determination of As species in human samples may lead to a further understanding of the mechanism of toxicity of As in humans. In Vietnam, the issue of arsenic contamination in groundwater and exposure by human consuming arsenic-contaminated water has become a matter of great concern and has been extensively investigated in recent years [5]. While most of the studies have examined As in groundwater, rice, and human urine and hair [5–9], very few information available about As species in paired human blood and urine samples from Vietnam has been reported. Development and validation of a suitable analytical method for determination of As species in different types of human samples such as blood and urine will be useful for the large-scale monitoring of human exposure to As in Vietnam.

Recently, many analytical methods have been developed for speciation analysis of arsenic compounds such as high-performance liquid chromatography in combination with atomic fluorescence spectrometry via hydride generator (HPLC-HG-AFS) [10], gas chromatography combination with tandem mass spectrometry (GC-MS/MS) [11], gas chromatography coupling with atomic emission spectrometry [12, 13], high-performance liquid chromatography in combination with atomic absorption spectrometry via hydride generator using online photoreactor (HPLC-UV-HG-AAS) [10], and high-performance liquid chromatography in combination with inductively coupled plasma mass spectrometry (HPLC-ICP-MS). Because of the natural physiochemical properties of arsenic species, anion-exchange chromatography in combination with ICP-MS becomes one of the most popular methods for separation of main arsenic species. Liquid chromatography in combination with molecular mass spectrometry has also been introduced for speciation analysis of arsenic compounds, especially in case of nontargeted analysis [14, 15]. In this study, for the first time, we investigated a method for determination of five arsenic species including arsenobetaine (AsB), dimethylarsinic acid (DMA), monomethylarsonic acid (MMA), arsenite (As^III^), and arsenate (As^V^) in urine and serum samples from Vietnam using HPLC with an anion-exchange column and ICP-MS, as a detector. The method was validated and then applied to analyze five As species in 17 paired human urine and serum samples from Hanoi, Vietnam.

## 2. Materials and Methods

### 2.1. Reagents and Standards

Asenobetaine (purum, pure form of analysis), arsenic(III) oxide (reagent plus), cacodylic acid (free acid, analytical standard), disodium methyl arsonate hexahydrate (analytical standard), and sodium arsenate dibasic heptahydrate were collected from Sigma-Aldrich (Singapore). Single stock solutions (1000 *µ*g·mL^−1^ as arsenic) were prepared by dissolving an extract amount of solid standard materials in appropriate deionized water and kept at polypropylene plastic tube at −20°C. Quantification of single standards was performed by comparison with commercially available arsenic standard for ICP-MS (TraceCERT, Sigma-Aldrich, Singapore) by utilizing flow injection ICP-MS measurement. Single stock solutions were stable at least 6 months at such conditions. Working solutions (from 0.5 to 100 ng·mL^−1^ as arsenic) were daily prepared by dilution of stock solutions in deionized water. Ammonium carbonate (metal analytical specification of Pharmacopeia of European), ammonium hydroxide (trace metals basis), methanol (LC-MS grade), and trichloroacetic acid were purchased from Sigma-Aldrich (Singapore). Mobile phase was prepared by dissolving appropriate amounts of ammonium carbonate and ethylenediaminetetraacetic acid disodium salt dihydrate (Na_2_EDTA) in a polypropylene bottle using deionized water and adjusted to given pH by adding concentrated ammonium hydroxide dropwise. A 2215 Hanna Instruments pH meter (Woonsocket, USA) was used for pH measurement. Mobile phase was degassed by the online vacuum degassing device of the LC 10A HPLC system. Mobile phase was stored in the plastic bottle, and fresh solution was prepared weekly. Deionized water (18.2 MΩ·cm) from ultrapure water system (Labconco, USA) was used for preparation of all standards and mobile phase.

#### 2.1.1. Sample Collection

Blood and urine samples were collected from 17 different patients in the No. 198 General Police Hospital, Hanoi, Vietnam. Blood samples were taken with disposable needles inserted into plastic tubes (Tube Traite, NJ, US) after clotting of the sera. After that, collected sera samples were kept at 4°C and then transported to the laboratory immediately within a day; other sera samples were stored at −18°C. The urine samples were taken in a plastic tube (Tube Traite, 50 mL, NJ, USA) and then stored at 4°C for storage and transported to the laboratory. Samples were stored at −18°C and analyzed within one month.

#### 2.1.2. Total As Determination

The urine and serum samples were digested in acidic condition in a microwave oven for total As determination. In brief, 0.1 mL of urine or serum was poured into microwave cell, 2 mL concentrated nitric acid (Suprapure, Merck, Singapore) and then 1 mL H_2_O_2_ (Merck, Singapore) was added and digested at 80% power of the Anton PARR (Graz, Austria) microwave oven. After digestion, the clear solution was transferred to precleaned 15 mL polypropylene tube and deionized water was added to 5 mL. These solutions were subjected to total arsenic analysis by ICP-MS. Fish-based matrix certified reference material (DORM III from National Research Council, Canada and BRC 211 from Institute for Reference Materials and Measurements, Belgium) was used for quality control of total arsenic analysis. Practically, the number of quality control and blank samples accounted for 20% of the number of total samples subjected for total analysis.

#### 2.1.3. Arsenic Speciation Analysis

For serum samples, protein in the serum sample was precipitated as follows: 400 *µ*L of serum sample was accurately weighed, and 500 *µ*L of 25% of trichloroacetic acid was added and then treated with 50 *µ*L of acetonitrile and 50 *µ*L deionized water, followed by being vortexed for 120 s. The mixture was then centrifuged at 10000×*g* for 10 min at 4°C. Clear aliquots of the supernatant were injected into HPLC-ICP-MS system.

For urine samples, a tenfold dilution of the samples was carried out with a mixture of deionized water and methanol (9/1, v/v). The mixture was then filtered via 0.45 *µ*m cellulose membrane and transferred to the LC plastic vial. This solution was subjected to HPLC-ICP-MS analysis.

### 2.2. Anion-Exchange Chromatography and Inductively Coupled Plasma Mass Spectrometry

Anion-exchange chromatography (AEC) analysis was carried on a LC 10A system (Shimadzu, Kyoto, Japan) including quaternary pump, online degasser, column oven, and system control unit. Arsenic species were separated on a Hamilton PRP-X100 anion-exchange column (250 × 2.1 mm × 10 *µ*m particle size) with an associated guard column (30 × 2.1 mm × 10 *µ*m particle size). Samples and standard solutions were injected via a 100 *µ*L sample loop by using a plastic syringe with silica-coated needle. Temperature of the column was kept at 30°C during chromatographic separation. Arsenic compounds were separated by employing a gradient of mixture of 5 mM (NH_4_)_2_CO_3_, pH 9.0 (adjusted by using NH_4_OH), 0.05% Na_2_EDTA (Chanel A) and 50 mM (NH_4_)_2_CO_3_, pH 9.0 (adjusted by using NH_4_OH), 0.05% Na_2_EDTA (Chanel B). The program of the mobile phase was set up as follows: the concentration of (NH_4_)_2_CO_3_ was 5 mM for 2 min followed by a linear increase to 50 mM in 10 min. After keeping at 50 mM for 5 min, the concentration of (NH_4_)_2_CO_3_ was then decreased to 5 mM in 1 min and kept for 9 min for re-equilibration of the column for the next injection. The flow rate of mobile phase was kept constant at 400 *µ*L·min^−1^ during chromatographic separation. Total time for separating of arsenic species was 25 min. A Perkin Elmer ELAN 9000 ICP-MS system (Perkin Elmer Sciex, Penlivia, Canada) was used as a specific elemental detector. The identity of arsenic species was assessed via comparison of retention times of single standards on the PRP-X100 column and tracer of arsenic ion on an ICP-MS detector. The operating conditions of ICPMS were controlled via using daily tuning solution. Optimized operating conditions for detection of arsenic by ICP-MS were performed via direct infusion by using 100 ng·mL^−1^ arsenic standard solution (Merck, Singapore) in acidic solution (2% HNO_3_). 500 mm PEEK tubing was used to connect the LC column and ICP-MS detector. The optimized operating conditions of AEC-ICP-MS are indicated in Table 1.

AEC-ICP-MS chromatogram was generated and exported via Thermo Xcalibur Version 4.0 (Thermo Fisher Scientific Inc, Bremen, Germany). Qualitative and quantitative browsers were used for integration and quantification of all chromatograms. Quantification of arsenic species in real samples was carried out by matrix-matched external calibration curves. Peak area was taken into account for quantification of target analytes.

## 3. Results and Discussion

### 3.1. Separation of Five Species of Arsenic on PRP-X100 Strong Anion-Exchange Column

For separation of five arsenic species, many studies used HPLC-ICP-MS as an effective method. Because of ionic compounds, either anion-exchange chromatography or ion pair chromatography has been employed for speciation of arsenic compounds. However, most of the mobile phases including phosphate buffer for anion-exchange chromatography or carbon rich mobile phase for reversed phase chromatography were used. As a major drawback, carbon build up and ion suppression commonly affected mobile phases in case of ion pair chromatography or influenced the physical properties of the sample introduction devices in case of anion-exchange chromatography employing high concentration of buffer, e.g., phosphate buffer. In this study, we developed the method from Peng and Wang et al. [15, 16] with some modifications. The mobile phase including ammonium carbonate, methanol, and ethylenediaminetetraacetic acid disodium salt (Na_2_EDTA) was adjusted to pH 9 and used for separation of five arsenic species.

**Figure 1:**
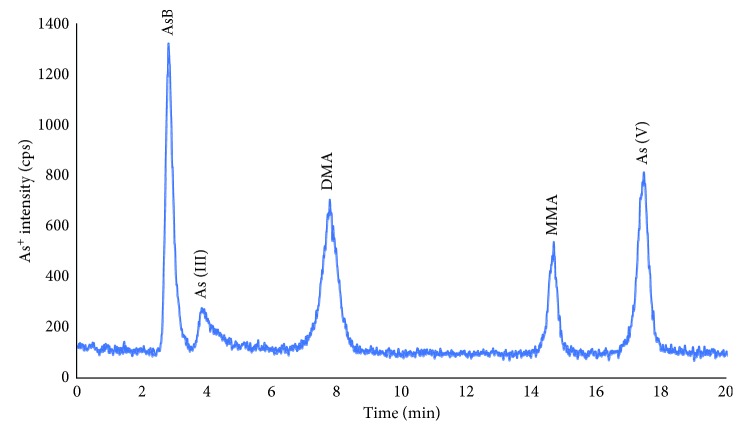
Chromatogram of five arsenic species (concentration of 2 ng·mL^−1^ according to As for each form) on the Hamilton PRP-X100 strong anion-exchange column using (NH_4_)_2_CO_3_, Na_2_EDTA, and MeOH as the mobile phase.

Adding Na_2_EDTA to mobile phase in order to obtain better peak shape has previously been reported in several studies of arsenic speciation. It can be explained by the formation of complex between EDTA and metal ions in sample matrices or stationary phase surface. The presence of EDTA is of great importance when the concentration of iron and alkaline metals (calcium, magnesium, etc.) is high, especially in environmental and biological samples [17]. Furthermore, the addition of Na_2_EDTA has demonstrated that it prevented the loss of the arsenic compounds during the chromatographic separation, especially arsenite [18]. Therefore, in this study, 0.05% EDTA (m/v) was added to the mobile phase and the concentration was kept constant for further experiments. In addition, the organic modifiers in mobile phase such as methanol and ethanol have been reported as improvement reagents for separation efficiency and analytical signal in anion-exchange chromatography [19–21]. Thus, four different concentrations of methanol in the mobile phase (0, 2, 5 and 10% v/v) were also investigated. The optimum concentration of methanol in the mobile phase was found to be 5% (v/v) for both separation efficiency and sensitivity. This phenomenon has also been confirmed in our previous work [22]. Therefore, such conditions were kept for the further experiments. In these experimental conditions, the interconversion among arsenic species was not observed (confirmation by injection of single standard versus time). In addition, all five arsenic species were baseline separated. The chromatogram of five arsenic species that obtained at the optimum condition is depicted in Figure 1.

**Figure 2:**
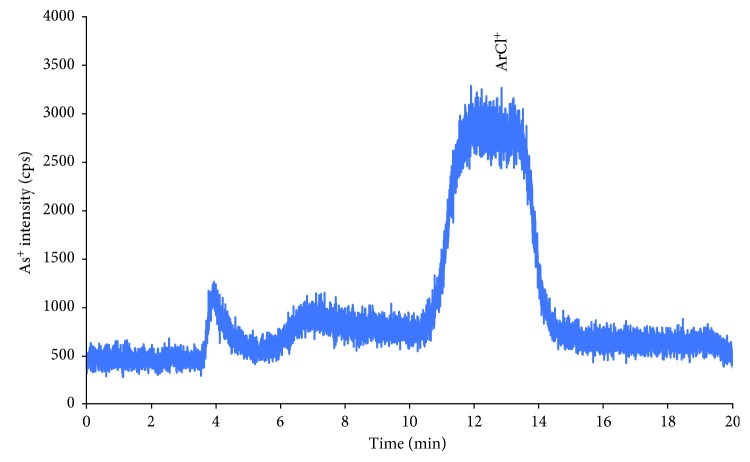
The chromatogram of 100 *µ*g·mL^−1^ of chloride in deionized water on the Hamilton PRP X100 strong anion-exchange column. Other experimental conditions are listed elsewhere.

As can be seen in Figure 1, all targeted arsenic species were baseline separated except a pair of AsB and As(III) (resolution between two compounds was 1.45). However, the resolution between AsB and As(III) was not affected on the quantification of As (III) in human serum and urine because of the extremely low concentration of As(III) in real sample. For As(III) in the real samples, the peak height was used for quantification of this compounds instead of the peak area. The isobaric mass of polyatomic interferences, which can come from the argon-based plasma such as ^40^Ar^35^Cl^+^, ^38^Ar^37^Cl^+^, and so on [23], is also a main drawback of quadrupole mass analyzer. To overcome the polyatomic interference in arsenic measurement, either dynamic reaction/collision cell or high-resolution ICP-MS was employed. In this study, for the assessment of mass interference on the arsenic measurement, 100 *µ*g·mL^−1^ of chloride was prepared in deionized water and injected on the HPLC-ICP-MS at the above condition. The chloride ion was eluted after DMA with low intensity. In addition, the peak of chloride ion was separated far from all arsenic species peaks in these chromatographic conditions (chromatogram in Figure 2). It should be noted that high chloride matrices such as human urine sample is important for arsenic speciation. The spiked experiments of chloride in real sample matrices (urine and serum) were also carried out, and the result showed that there was no statistically significant effect of chloride ion on the quantification of arsenic species in such matrices.

Table 2 lists the chromatographic characteristics of the optimized method, i.e., retention time, chromatographic resolution, and peak width at 50% peak height calculated according to European Pharmacopeia. Other critical parameters of the developed method are repeatability of the retention time and analytical signal (peak area). For the assessment of stability of the retention time and peak area, three independent solutions of five arsenic species were freshly prepared in deionized water and injected into the HPLC-ICP-MS system. The stability of retention time was achieved in the range of 0.2–3.0% (short term, *n* = 3) and 0.4–10% (long term, 20 hours) for all AsB, As(III), MMA, DMA, and As(V). The experimental results demonstrated that the excellent repeatability of retention time was achieved for separation of arsenic species via anion-exchange chromatography. After approximately 100 injections of samples (especially real samples), the column was regenerated by back flush using a solvent as the suggestion of the manufacturer. The repeatability of the analytical signal is a parameter, which has a significant contribution on the uncertainty of measurement. The repeatability of peak areas of all arsenic species was assessed by injecting an arsenic standard mixture solution at different times. Relative standard deviations of peak areas of five arsenic compounds were in range of 2–10% and 4–16% for short-term and long-term stability, respectively. It was worthy noted that the good reparability of peak areas was achieved in this study.

### 3.2. Analytical Figure of Merits

For assessment of linearity range, limit of detection (LOD), and limit of quantification (LOQ), six independent solutions with concentration range of 4 to 50 ng·mL^−1^ (except As (III)) were prepared in deionized water and injected triplicates on the HPLC-ICP-MS system. Peak areas of five arsenic species were taken into account as linearity functions of respective arsenic species concentrations. The LOD and LOQ were calculated by three times and ten times of signal to noise ratio as guideline of the US Food and Drug Administration [24, 25]. The results are shown in Table 3.

As can be seen from Table 3, a very good relation between analytical signal and concentration of arsenic species was obtained (*R*^2^ > 0.999 in all cases). The LOD and LOQ revealed that this method had enough sensitivity for direct quantification of arsenic species in human urine and serum samples. The LOD and LOQ in this study are in good agreement with some previously published papers [26, 27].

### 3.3. Extraction Efficiency of Arsenic Species in Human Urine and Serum Samples

In this study, urine samples were prepared by dilution in the mixture of methanol and deionized water (1 : 9, *v* : *v*). Dilution of urine samples was also tested by using a mixture of mobile phase (pH 9.0) and methanol. However, interconversion among arsenic species, especially the conversion of arsenite into arsenate, was observed. This phenomenon has been also confirmed by Verdon et al. [28]. Recovery of arsenic species was carried out by spiking experiments using pooled urine samples. The recoveries of AsB, As(III), MMA, DMA, and As(V) in pooled urine sample were 112, 115, 91, 93, and 97.5%, respectively. The interconversion among arsenic species was not observed in this solvent. In addition, the retention time shift was not observed in such dilution solvent. Therefore, the mixture of deionized water and methanol was chosen for the dilution of urine sample.

For serum samples, several sample preparation procedures such as alkaline digestion and protein precipitation in combination with HybirdSPE cartridge (Sigma-Aldrich, Singapore) were tested. However, recovery of arsenic species was either of low efficiency or interconversion among species, especially in case of arsenite. Therefore, the removal of serum protein by precipitation with acetonitrile and trichloroacetic acid was chosen for isolation of arsenic species. For the assessment of the selected sample preparation procedure, the pooled serum sample was prepared by mixing six serum samples. Pooled serum sample was prepared as described in the aforementioned section. The recovery of arsenic species in pooled serum samples was performed via spiking experiments. The recoveries of AsB, As(III), MMA, DMA, and As(V) were found to be 105, 94, 124, 137, and 139%, respectively. The low recovery of As(III) and high recovery of As(V) could be attributed to either interconversion during sample preparation or contaminated during sample preparation. In addition, ionization suppression/enhancement effects, especially in the real sample, are other causes that also affected the recovery. However, the recovery of arsenic species at such concentration levels could be acceptable for monitoring of chemical exposure on human health [29].

### 3.4. Arsenic Speciation Analysis in Vietnamese Human Urine and Serum Samples

**Figure 3:**
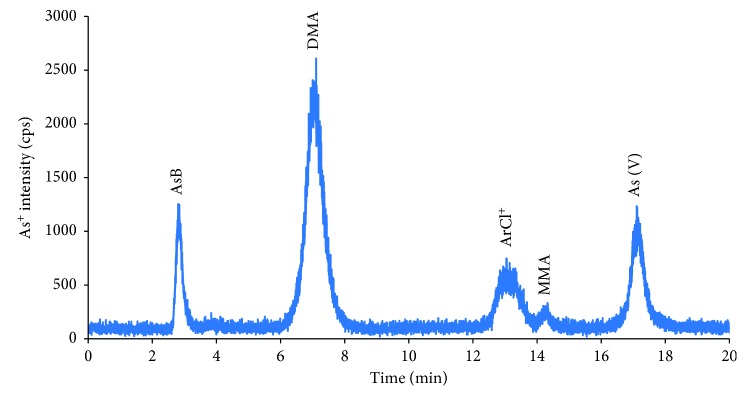
Chromatogram of arsenic species in serum sample. Other experimental conditions are mentioned in text.

**Figure 4:**
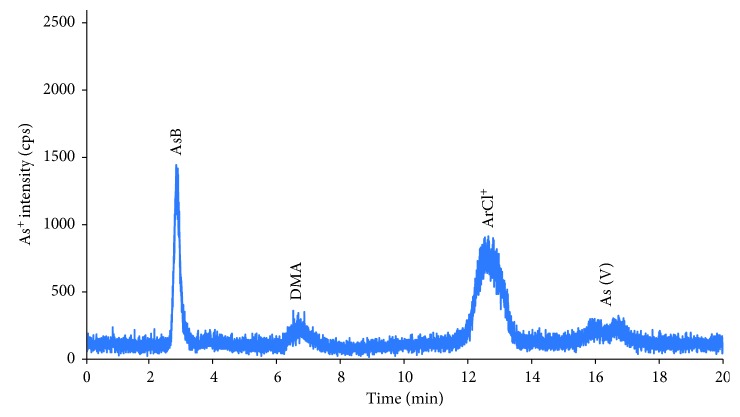
Chromatogram of arsenic species in urine sample. Other experimental conditions are mentioned in text.

For application of the proposed method, 17 pairs of urine and serum samples that were collected in local hospital were analyzed as aforementioned procedure. The chromatograms of arsenic compounds in serum and urine samples are indicated in Figures 3 and 4, respectively.

The average concentrations of arsenic species in serum and urine samples are demonstrated in Table 4. All arsenic species were found in the serum and urine samples. However, major arsenic species in urine samples were AsB and DMA; meanwhile, DMA, As(III), and AsB were the most abundant ones in serum samples. In addition, the total concentrations of inorganic arsenic compounds in both serum and urine were calculated by the sum of arsenite and arsenate. The concentration of inorganic arsenic compounds ranged from 1.84 to 8.0 ng·mL^−1^. It implies that main sources of arsenic exposure to humans mostly come from water and rice [30, 31].

## 4. Conclusions

In this study, compatible mobile phase has been introduced for speciation analysis of five main arsenic compounds via strong anion-exchange chromatography in combination with inductively coupled plasma mass spectrometry as a specific elemental detector. Some critical parameters of analytical method such as LOD, LOQ, repeatability, and extraction efficiency have been systematically investigated and implemented. The sensitivity of the introduced method was high enough for direct quantitation of five arsenic compounds in human serum and urine samples. The proposed method has been successfully applied for analysis of 17 pairs of urine and serum samples. Our result indicates that the main exposure of arsenic compounds is rice and water through a diet pathway. The developed method will be applied for human serum and urine samples that were collected from people living in the highly contaminated arsenic areas in Northern of Vietnam to evaluate the status of arsenic contamination and human exposure.

## Figures and Tables

**Table 1:** Operating conditions of anion-exchange chromatography (AEC) combined with inductively coupled plasma mass spectrometry (ICP-MS) for speciation analysis of arsenic compounds.

Anion-exchange chromatography operating conditions	
Pump	Quaternary pump LC 10 A (Shimadzu, Japan) including online degasser, column oven, and system control unit
Column	Hamilton PRP-X100 anion-exchange column (250 × 2.1 mm × 10 *µ*m particle size) with an associated guard column
Column temperature	30°C
Mobile phase	Chanel A: 5 mM (NH_4_)_2_CO_3_, pH 9.0, 0.05% Na_2_ EDTA
	Chanel B: 50 mM (NH_4_)_2_CO_3_, pH 9.0, 0.05% Na_2_ EDTA
	Chanel C: MeOH
	Elution mode gradient
Flow rate of mobile phase	400 *µ*L·min^−1^
Injection volume	100 *µ*L

ICP-MS operating conditions	
ICP-MS system	ELAN 9000 (PerkinElmer Sciex, Penlivia, Canada)
RF powder	1300 W
Plasma gas flow	16 L·min^−1^
Auxiliary gas flow	1.25 L·min^−1^
Nebulizer gas flow	0.9 L·min^−1^
Nebulizer	Cross-flow
Spray chamber	PFA double pass
Monitored ion	As^+^ (*m*/*z* 74.92)
Dwell time	100 ms
Measurement mode	Peak hopping

**Table 2:** Characteristics of the chromatography separations of arsenic compounds on the Hamilton PRP-X100 anion-exchange column using conditions given in the experimental sections.

No	Analytes	Abbr.	*t*_R_ (min)	*R*	*W*_1/2_ (min)
1	Arsenobetaine	AsB	2.82	—	0.24
2	Arsenite	As(III)	3.84	1.45	0.46
3	Dimethylarsinic acid	DMA	7.78	3.51	0.36
4	Monomethylarsonic acid	MMA	14.7	4.67	0.25
5	Arsenate	As(V)	17.5	2.53	0.40

*t*_R_: retention time; *R*=2 *∗*((*t*_*R*,  *i*+1_ − *t*_*R*,  *i*_)/(*W*_*i*+1_+*W*_*i*_)): chromatographic resolution; *W*_1/2_: width at 50% peak height.

**Table 3:** Characteristics of analytical figure of merits of arsenic species quantified by HPLC-ICP-MS.

Analytes	Linearity range (ng·mL^−1^)	Regression coefficient	LOD (ng·mL^−1^)	LOQ (ng·mL^−1^)	LOD^*∗*^ (pg)	LOQ^*∗*^ (pg)
AsB	3–100	0.9999	0.38	1.20	38	120
As(III)	4–50	0.9999	1.5	5.0	150	500
DMA	2–50	0.9997	0.35	1.20	35	120
MMA	4–50	0.9998	0.40	1.3	40	130
As(V)	4–50	0.9999	0.27	0.91	27	91

^*∗*^Absolute limit of detection and limit of quantification.

**Table 4:** Average concentrations of arsenic species in serum and urine.

Sample	Concentrations of As species (ng·mL^−1^)						
	AsB	As(III)	DMA	MMA	As(V)	Total iAs^a^	Total As^b^
*Serum*							
Male (*n* = 7)	6.93 (1.63–19.8)	7.31 (1.5–22.0)	6.01 (0.35–11.23)	0.46 (0.4–0.8)	0.27 (0.27–0.27)	7.58 (1.77–22.3)	20.5 (6.4–26.5)
Female (*n* = 10)	6.64 (1.09–17.7)	7.20 (1.5–21.6)	10.0 (0.35–39.6)	1.46 (0.4–0.98)	0.80 (0.27–5.54)	8.00 (1.77–27.14)	24.9 (13.7–40.5)

*Urine*							
Male (*n* = 7)	2.26 (0.36–4.28)	1.50 (1.5–1.50)	2.16 (0.47–3.80)	0.97 (0.15–1.52)	0.56 (0.09–1.3)	2.06 (1.59–2.8)	60.6 (22.7–99.6)
Female (*n* = 10)	1.85 (0.25–5.20)	1.50 (1.5–1.50)	1.77 (0.43–3.66)	0.91 (0.36–1.35)	0.34 (0.27–0.83)	1.84 (1.77–2.33)	46.0 (9.60–106)

^a^Total concentrations of inorganic arsenic species were defined by the sum of As(III) and As(V); ^b^total concentrations of arsenic were determined by microwave digestion and ICPMS measurement.

## Data Availability

The data in the manuscript can be accessed at Faculty of Chemistry, VNU-University of Science, Vietnam National University, Hanoi, 19 Le Thanh Tong, Hoan Kiem, Hanoi 10000, Vietnam; Department of Analytical Chemistry, School of Chemical Engineering, Hanoi University of Science and Technology, 1 Dai Co Viet, Hai Ba Trung, Hanoi 100000, Vietnam; and National Institute of Hygiene and Epidemiology, 1 Yersin, Hai Ba Trung, Hanoi 100000, Vietnam. There are some restrictions on data access due to lack of connection among the above three faculties. Three faculties belong to different kinds of universities and Institutes.
